# Rush Medical College

**Published:** 1867-08

**Authors:** 


### *Rush Medical College.*

The new building is rapidly progressing. There is now not the slightest doubt that it will be fully completed within the time specified in the contracts. Its stately proportions and convenient arrangements already attract large attention from visitors of the city.

As one looks over the subject, it is a matter of increasing surprise that the College could have gained such great prosperity whilst so badly cabinned in the old building. A large concourse is confidently anticipated at the opening of the ensuing session.

